# Cerenkov luminescence imaging of interscapular brown adipose tissue using a TSPO-targeting PET probe in the UCP1 ThermoMouse

**DOI:** 10.7150/thno.74828

**Published:** 2022-08-29

**Authors:** Seok-Yong Lee, Ho Rim Oh, Young-Hwa Kim, Sung-Hwan Bae, Yongseok Lee, Yun-Sang Lee, Byung Chul Lee, Gi Jeong Cheon, Keon Wook Kang, Hyewon Youn

**Affiliations:** 1 Department of Nuclear Medicine, Seoul National University College of Medicine, Seoul, Republic of Korea; 2 Department of Biomedical Sciences, Seoul National University Graduate School, Seoul, Republic of Korea; 3 Cancer Research Institute, Seoul National University College of Medicine, Seoul, Republic of Korea; 4 Cancer Imaging Center, Seoul National University Hospital, Seoul, Republic of Korea; 5 Radiation Medicine Research Institute, Medical Research Center, Seoul National University College of Medicine, Seoul, Republic of Korea; 6 Department of Nuclear Medicine, Seoul National University Bundang Hospital, Seongnam, Republic of Korea; 7 Center for Nanomolecular Imaging and Innovative Drug Development, Advanced Institutes of Convergence Technology, Seoul National University, Suwon, Republic of Korea

**Keywords:** Interscapular brown adipose tissue, UCP1, TSPO, PET, Cerenkov luminescence imaging

## Abstract

**Rationale:** [^18^F]fluorodeoxyglucose-positron emission tomography ([^18^F]FDG-PET) has been widely used as an imaging technique to measure interscapular brown adipose tissue (iBAT) activity. However, it is challenging to obtain iBAT-specific images using [^18^F]FDG-PET because increased uptake of [^18^F]FDG is observed in tumors, muscle, and inflamed tissues. Uncoupling protein 1 (UCP1) in the mitochondrial membrane, a well-known molecular marker of BAT, has been proposed as a useful BAT imaging marker. Recently, the UCP1 ThermoMouse was developed as a reporter mouse for monitoring UCP1 expression and investigating BAT activation. In addition, Translocator protein-18 kDa (TSPO) located in the outer mitochondrial membrane is also overexpressed in BAT, suggesting that TSPO-targeting PET has potential for iBAT imaging. However, there are no studies monitoring BAT using TSPO-targeting PET probes in the UCP1 ThermoMouse. Moreover, the non-invasive Cerenkov luminescence imaging (CLI) using Cerenkov radiation from the PET probe has been proposed as an alternative option for PET as it is less expensive and user-friendly. Therefore, we selected [^18^F]fm-PBR28-*d*_2_ as a TSPO-targeting PET probe for iBAT imaging to evaluate the usefulness of CLI in the UCP1 ThermoMouse.

**Methods:** UCP1 ThermoMouse was used to monitor UCP1 expression. Western blotting and immunohistochemistry were performed to measure the level of protein expression. [^18^F]fm-PBR28-*d*_2_ and [^18^F]FDG were used as radioactive probes for iBAT imaging. PET images were acquired with SimPET, and optical images were acquired with IVIS 100.

**Results:** UCP1 ThermoMouse showed that UCP1 and TSPO expressions were correlated in iBAT. In both PET and CLI, the TSPO-targeting probe [^18^F]fm-PBR28-*d*_2_ was superior to [^18^F]FDG for acquiring iBAT images. The high molar activity of the probe was essential for CLI and PET imaging. We tested the feasibility of TSPO-targeting probe under cold exposure by imaging with TSPO-PET/CLI. Both signals of iBAT were clearly increased after cold stimulation. Under prolonged isoflurane anesthesia, TSPO-targeting images showed higher signals from iBAT in the short-term than in long-term groups.

**Conclusion:** We demonstrated that TSPO-PET/CLI reflected UCP1 expression in iBAT imaging better than [^18^F]FDG-PET/CLI under the various conditions. Considering convenience and cost, TSPO-CLI could be used as an alternative TSPO-PET technique for iBAT imaging.

## Introduction

Interscapular brown adipose tissue (iBAT) is an organ that plays an important role in maintaining heat production and energy homeostasis in mammals 1, 2. Thus, impairment of the heat-generating function of iBAT contributes to metabolic diseases such as obesity. Various imaging modalities for iBAT have been developed 3, 4, such as positron emission tomography (PET) using [^18^F]fluorodeoxyglucose ([^18^F]FDG) that can visualize iBAT under cold or chemical stimulation conditions 5-9. However, [^18^F]FDG is not compatible with iBAT-specific imaging due to its strong absorption in malignant cancers, inflammation, or during muscle activity 10. Furthermore, iBAT imaging with [^18^F]FDG-PET needs to consider endocrine factors, such as individual differences in insulin sensitivity and epigenetic programs, which cannot be explained by glucose uptake 11, 12.

Uncoupling protein 1 (UCP1) is most abundantly expressed in the mitochondrial inner membrane and also overexpressed in brown adipose tissue (BAT) and used as its biomarker. UCP1 uncouples the respiratory chain from oxidative phosphorylation and ATP synthesis enabling energy use 13, 14. The UCP1 ThermoMouse was recently developed to monitor its expression 15. Translocator protein-18 kDa (TSPO) is a five transmembrane domain protein located on the mitochondrial outer membrane 16 and used to characterize many cancers and inflammations 17. Various radioligands targeting TSPO have been developed as PET probes for imaging neuroinflammation 18-22. TSPO is abundantly expressed in mitochondria and has recently emerged as a potential imaging biomarker for iBAT because of its enhanced expression in iBAT compared to the inguinal white adipose tissue (iWAT) 23, 24. Successful iBAT-PET imaging using [^11^C]PBR28 that targets TSPO has been reported 25. Our group also reported that deuterium-substituted [^18^F]fluormethyl-PBR28-*d*_2_ ([^18^F]fm-PBR28-*d*_2_) overcomes the metabolic stability and adverse short half-life of [^11^C] (T_1/2_ = 20.36 min) 26.

Cerenkov luminescence imaging (CLI) is a new optical imaging technique that captures the emission of electromagnetic radiation from β^+^ or β^-^ decay of radionuclides, such as [^18^F] and [^131^I], in the medium. Cerenkov radiation (CR) is observed when a charged particle moving through a medium is faster than the phase velocity of light in that medium 27, 28. With optical imaging equipment, CLI can be acquired with PET probes. Although CLI techniques for imaging iBAT using [^18^F]FDG have been tested 29-31, most studies focused on monitoring the biodistribution of [^18^F]FDG based on anatomical location without comparing the expression of UCP1, a biomarker of BAT. Currently, there are no reports of PET and CLI images using nuclear medicine probes such as [^18^F]FDG or [^18^F]fm-PBR28-*d*_2_ based on bioimaging of UCP1 expression, a biomarker of BAT. In this study, we compared UCP1 expression and PET signals from iBAT using [^18^F]FDG or [^18^F]fm-PBR28-*d*_2_ imaging in UCP1 ThermoMouse and discussed the possibility of CLI as an alternative option to PET.

## Results

### UCP1 ThermoMouse is useful for imaging in vivo UCP1 expression in the iBAT

**Figure 1:**
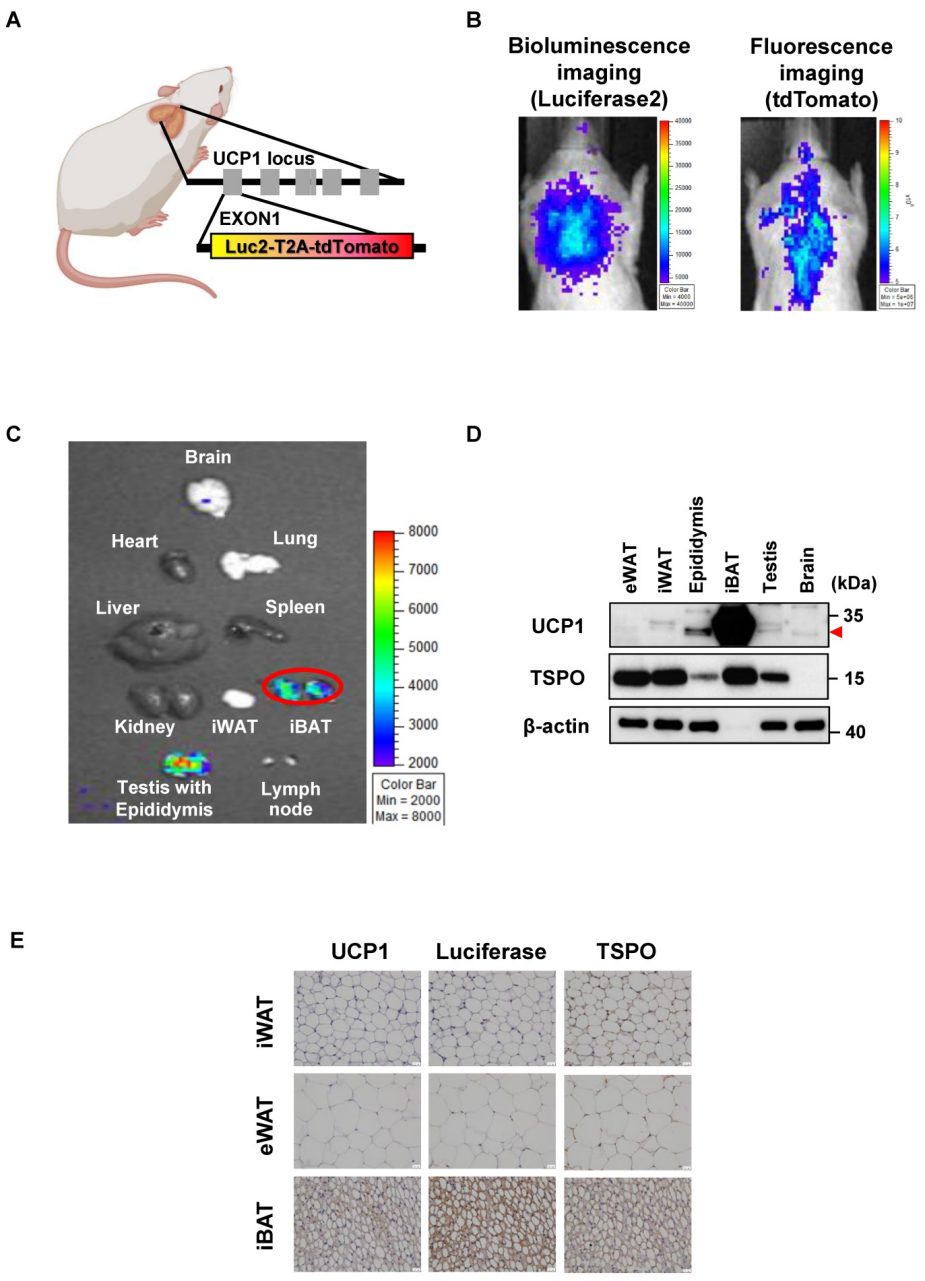
UCP1 ThermoMouse is useful for imaging *in vivo* UCP1 expression in the iBAT. (A) Design of reporter gene construct of UCP1 ThermoMouse. (B) Bioluminescence and fluorescence imaging of interscapular brown adipose tissue (iBAT). (C) Bioluminescence imaging (BLI) for monitoring UCP1 expression in mouse organs. (D) UCP1 and TSPO protein expression in different adipose tissues and organs by western blotting. (E) Immunohistochemical staining of UCP1, luciferase, and TSPO in inguinal white adipose tissue (iWAT), epididymal white adipose tissue (eWAT) and iBAT. All slides were counterstained with hematoxylin. (x400 magnification). Scale bar, 20 μm. Figure was created by BioRender.com**.**

UCP1 ThermoMouse is a reporter mouse for monitoring *in vivo* UCP1 expression generated by inserting the luciferase2-T2A-tdTomato cassette into the initiation codon of 98.6 kb bacterial artificial chromosome (BAC) containing the entire UCP1 locus 15. Thus, bioluminescence from luciferase2 (Luc2) and fluorescence from tdTomato under the control of UCP1 expression could be monitored (**Figure 1A**). The luciferase activities and fluorescence signals from UCP1 expression were observed in the iBAT region (**Figure 1B**). As displayed in **Figure 1C,** analysis of UCP1 expression in the whole body of the UCP1 ThermoMouse showed higher luminescence signals from iBAT and testis with epididymis than other tissues. It is thought that a shorter distance between the detector and the object exaggerates the signal in the optical image. Thus, the epididymis produces a relatively stronger BLI signal because it protrudes above the testis and is closer to the detector compared to the flat iBAT.

Since UCP1 is used as a BAT biomarker and the TSPO-targeting probe is considered an iBAT-specific imaging marker 25, we tested the correlation between UCP1 and TSPO expression in iBAT using western blotting and immunohistochemistry (IHC). UCP1 and TSPO expression in iBAT were significantly higher than in other tissues (**Figure 1D**). **Figure 1E** shows UCP1, luciferase, and TSPO expression in UCP1 ThermoMouse tissues, and demonstrates increased expression in iBAT. Although the expression of UCP1 was observed in the epididymis, epididymis and iBAT were quite separated in anatomical location. Thus, we concluded that the UCP1 ThermoMouse could be used to represent *in vivo* UCP1 expression in the iBAT.

### TSPO targeting probe, [^18^F]fm-PBR28-d_2_ is superior to obtaining iBAT imaging than [^18^F]FDG

**Figure 2:**
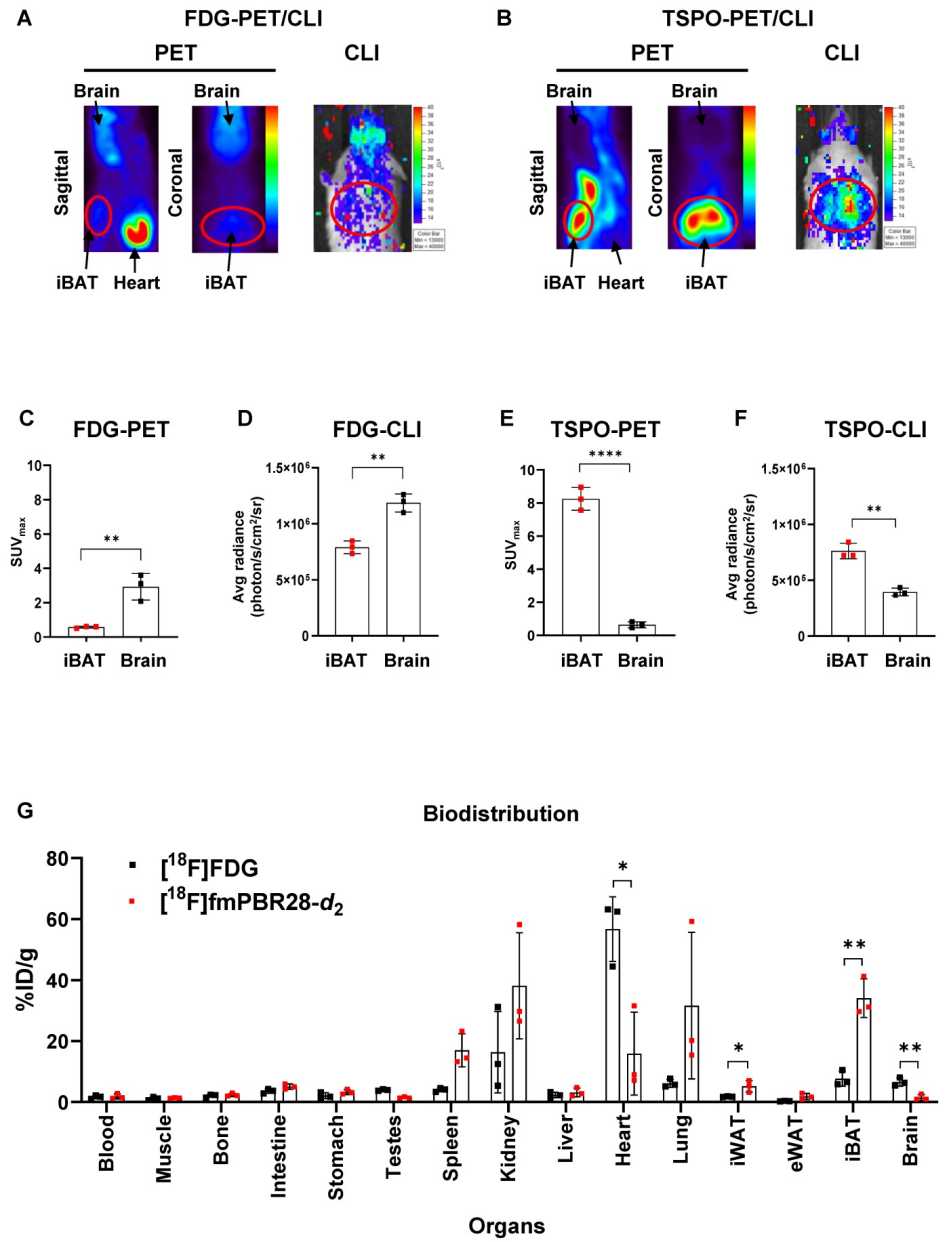
TSPO targeting probe, [^18^F]fm-PBR28-*d*_2_ is superior to obtaining iBAT imaging than [^18^F]FDG. (A) Representative [^18^F]FDG-PET and CLI images. (B) Representative [^18^F]fm-PBR28-*d*_2_-PET and CLI images. (C) Quantitative analysis of PET signals (SUV_max_ ratio for PET) from iBAT after [^18^F]FDG injection. (D) Quantitative analysis of CLI signals (Average radiance, photon/s/cm^2^/sr for CLI) from iBAT after [^18^F]FDG injection. (E) Quantitative analysis of PET signals (SUV_max_ ratio for PET) from iBAT after [^18^F]fm-PBR28-*d*_2_ injection. (F) Quantitative analysis of CLI signals (Average radiance, photon/s/cm^2^/sr for CLI) from iBAT after [^18^F]fm-PBR28-*d*_2_ injection. (G) Biodistribution of [^18^F]FDG or [^18^F]fm-PBR28-*d*_2_ in UCP1 ThermoMice. Data represent means ± SD (n =  3 per group). *P < 0.05, **P < 0.01, ****P < 0.0001.

We performed [^18^F]FDG-PET and TSPO-PET for iBAT imaging because significant uptake of [^18^F]FDG in the iBAT area under cold exposure in humans was previously reported in many studies 5-9. We tested the possibility of CLI as an alternative option to PET for iBAT imaging by comparing the relationship between luciferase reporter imaging of UCP1 expression and PET/CLI imaging of [^18^F]FDG or [^18^F]fm-PBR28-*d*_2_ in the UCP1 ThermoMouse model. Strong uptake [^18^F]FDG was observed in iBAT by PET and CLI imaging, but the [^18^F]FDG signal was also observed in the brain (**Figure 2A**). However, as evident from **Figure 2B,** PET and CLI of [^18^F]fm-PBR28-*d*_2_ targeting TSPO showed a strong signal in iBAT without its uptake in the brain. We quantitatively compared PET and CLI signals from [^18^F]FDG and [^18^F]fm-PBR28-*d*_2_. In PET imaging, [^18^F]FDG uptake in the brain was 5.08-fold higher than in iBAT (SUV_max_, “iBAT” *vs.* “brain”, 0.575 ± 0.058 *vs.* 2.929 ± 0.7742, ^**^P = 0.0063, **Figure 2C**). As expected, consistent with previous PET imaging results, CLI signal from [^18^F]FDG in the brain was 1.5-fold higher than in iBAT (Average radiance, photon/s/cm^2^/sr, “iBAT” *vs.* “brain”, 791000 ± 57026.3 *vs.* 1186667 ± 80829, ^**^P = 0.0023, **Figure 2D**). On the other hand, [^18^F]fm-PBR28-*d*_2_ uptake in iBAT was 12.92-fold higher than in the brain (SUV_max_, “brain” *vs.* “iBAT", 0.637 ± 0.182 *vs.* 8.246 ± 0.688, ^****^P < 0.0001, **Figure 2E**). Furthermore, the CLI signal from [^18^F]fm-PBR28-*d*_2_ in iBAT was 1.93-fold higher than in the brain (Average radiance, photon/s/cm^2^/sr, “brain” *s.* “iBAT”, 395000 ± 35085.6 *vs.* 762333.3 ± 68995.2, ^**^ P = 0.0012, **Figure 2F**). Next, we examined the biodistribution of [^18^F]FDG and [^18^F]fm-PBR28-*d*_2_ in the UCP1 ThermoMouse and detected significantly higher [^18^F]FDG uptake in the heart and brain compared to [^18^F]fm-PBR28-*d*_2_ (%ID/g, [^18^F]FDG *vs.* [^18^F]fm-PBR28-*d*_2_, 56.74 ± 10.6 *vs.* 15.88 ± 13.66, ^*^P = 0.014 for the heart; 6.62 ± 1.38 *vs.* 1.52 ± 0.96, ^**^P = 0.0063 for the brain). Notably, we found that [^18^F]fm-PBR28-*d*_2_ uptake in iBAT was significantly higher than [^18^F]FDG uptake (Percentage of injected dose per gram (%ID/g), [^18^F]FDG *vs.* [^18^F]fm-PBR28-*d*_2_, 7.68 ± 2.47 *vs.* 34.08 ± 6.32, ^**^P = 0.0025) (**Figure 2G and Table 1**).

### [^18^F]fm-PBR28-d_2_ reflects endogeneous UCP1 levels much better than [^18^F]FDG

**Figure 3:**
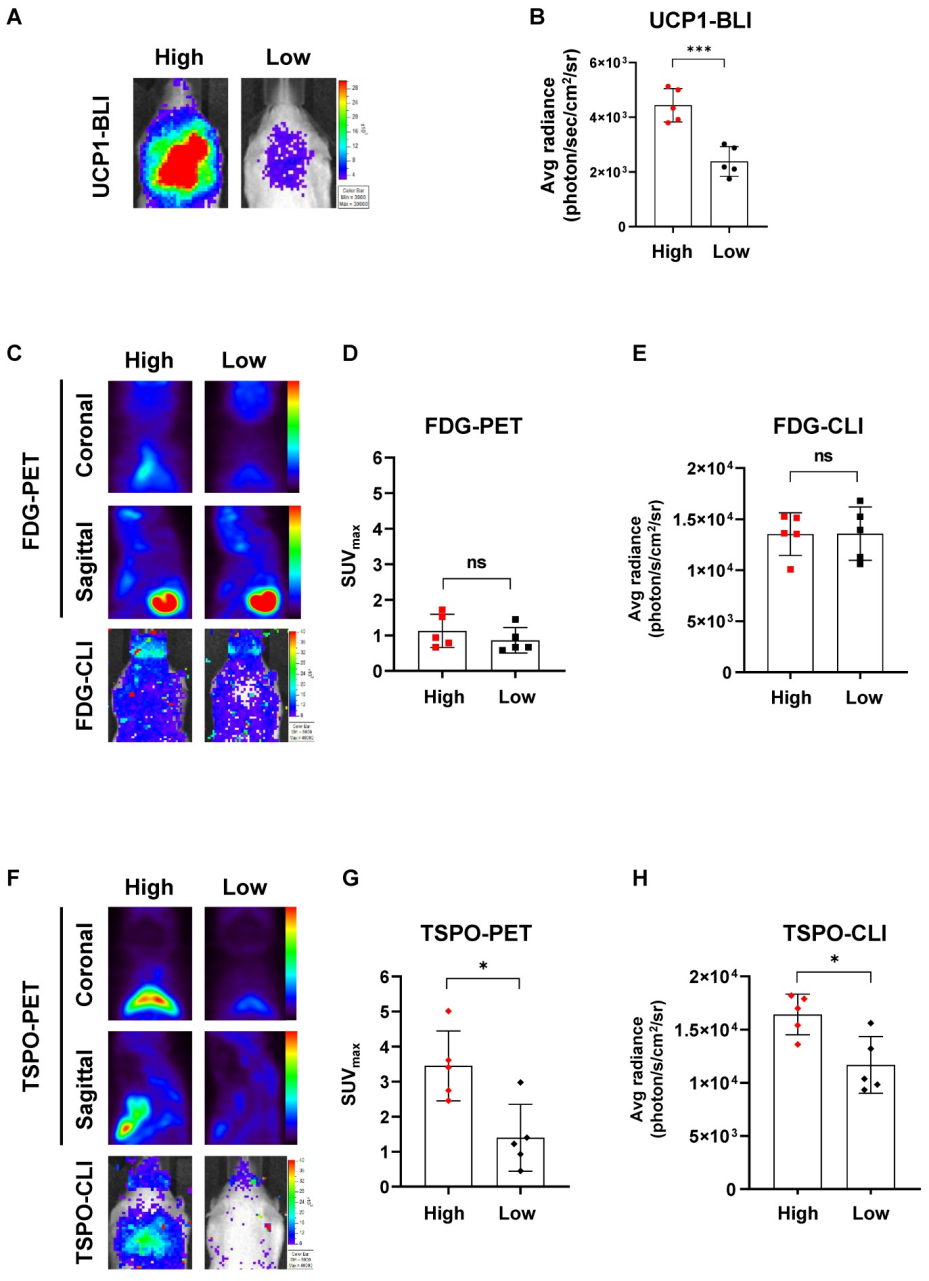
[^18^F]fm-PBR28-*d*_2_ reflects endogenous UCP1 levels much better than [^18^F]FDG. (A) Representative BLI in groups of ThermoMice with “high” or “low” endogenous UCP1 expression. (B) Quantitative analysis of BLI signals from iBAT between groups with “high” and “low” UCP1 expression. (C) Representative [^18^F]FDG-PET and CLI images in groups with “high” and “low” UCP1 expression. (D) Quantitative analysis of [^18^F]FDG-PET signals from iBAT in groups with “high” and “low” UCP1 expression. (E) Quantitative analysis of [^18^F]FDG-CLI signals from iBAT in groups with “high” and “low” UCP1 expression. (F) Representative TSPO-PET and CLI images in groups with “high” and “low” UCP1 expression. (G) Quantitative analysis of TSPO-PET signals from iBAT in groups with “high” and “low” UCP1 expression. (H). Quantitative analysis of TSPO-CLI signals from iBAT in groups with “high” and “low” UCP1 expression. Data represent means ± SD (n  =  5 per group). *P < 0.05, ***P < 0.001.

Because UCP1 expression generally varies between individual UCP1 ThermoMouse, we divided them into two groups based on the endogenous UCP1 levels after monitoring luciferase activity with BLI (**Figure 3A**). Subsequently, we compared PET and CLI images with [^18^F]FDG and [^18^F]fm-PBR28-*d*_2_ between the two groups. UCP1 expression, represented by the BLI signal, was 1.85-fold higher in the “high” group than in the “low” group (Average radiance, photon/s/cm^2^/sr, “high” *vs.* “low”, 4435.6 ± 609.5 *vs.* 2387 ± 542.83, ^***^P = 0.0005, **Figure 3B**). Then, we analyzed [^18^F]FDG-PET and [^18^F]FDG-CLI images and found no difference in [^18^F]FDG signals from PET and CLI between groups with different UCP1 levels (**Figure 3C**). Furthermore, no statistical significance was observed between groups of different UCP1 levels in quantitative analysis of [^18^F]FDG-PET (SUV_max,_ “high” *vs.* “low”, 1.12 ± 0.46 *vs.* 0.86 ± 0.36, not significant (ns), **Figure 3D**) and [^18^F]FDG-CLI (Average radiance, photon/s/cm^2^/sr, “high” *vs.* “low”, 13534 ± 2087.47 *vs.* 13578 ± 2613.71, ns, **Figure 3E**). However, as **Figure 3F** shows, [^18^F]fm-PBR28-*d*_2_ signals from TSPO-PET and TSPO-CLI were significantly higher in the “high” UCP1 group than in the “low” UCP1 group. Similarly, quantitative analysis of TSPO-PET and CLI showed that both signals were significantly higher in the “high” UCP1 group than in the “low” UCP1 group (SUV_max_, “high” *vs.* “low”, 3.47 ± 0.99 *vs.* 1.40 ± 0.95, ^*^P = 0.0104; average radiance, photon/s/cm^2^/sr, 16422 ± 1912.57 *vs.* 11673 ± 2655.12, ^*^P = 0.012 for CLI, **Figure 3G-H**). These data suggested that [^18^F]fm-PBR28**-***d*_2_ produced more specific images for iBAT than [^18^F]FDG.

### High molar activity is essential for acquiring TSPO-CLI as well as TSPO-PET on iBAT imaging

**Figure 4:**
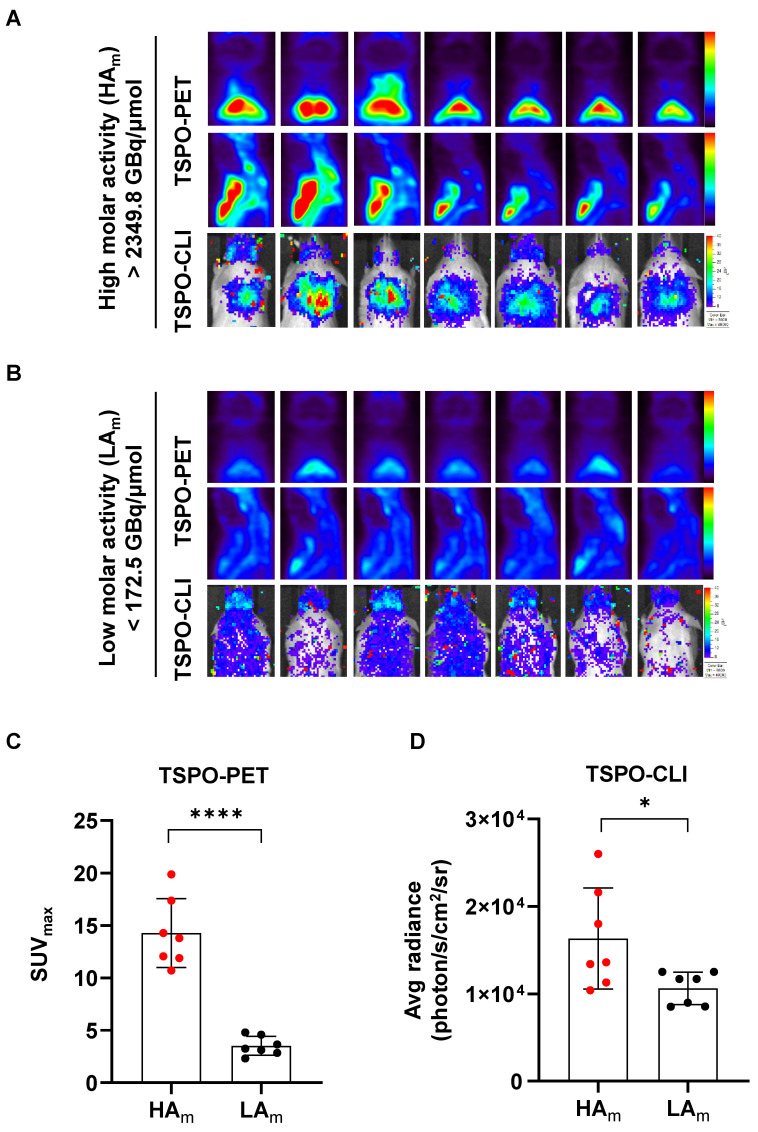
High molar activity is essential for acquiring TSPO-CLI as well as TSPO-PET of iBAT imaging. (A) Representative TSPO-PET and TSPO-CLI for iBAT images using [^18^F]fm-PBR28-*d*_2_ with high molar activity (“HA_m_”, more than 2349.8 GBq/μmol). (B) Representative TSPO-PET and TSPO-CLI for iBAT images using [^18^F]fm-PBR28-*d*_2_ with low molar activity (“LAm”, less than 172.5 GBq/μmol). (C) Quantitative analysis of the PET signals from [^18^F]fm-PBR28-*d*_2_ with high and low molar activity in iBAT images of UCP1 ThermoMice. (D) Quantitative analysis of CLI signals from [^18^F]fm-PBR28-*d*_2_ with high and low molar activity in iBAT images of UCP1 ThermoMice. Data represent means ± SD (n  = 7 per group). *P < 0.05, ****P < 0.0001.

Since our results showed that [^18^F]fm-PBR28**-***d*_2_ was better than [^18^F]FDG for iBAT imaging, we investigated conditions for generating better images of [^18^F]fm-PBR28**-***d*_2_-PET and CLI. Probes with high molar (or specific) activity generally contain fewer non-radiolabeled precursors competing with radiolabeled molecules, resulting in better images. Therefore, we investigated qualitative differences in iBAT images between probes with different molar activities of [^18^F]fm-PBR28**-***d*_2_ for TSPO-PET and CLI. As is evident from **Figure 4A,** PET and CLI signals of iBAT with the high molar activity probe (“HA_m_”, more than 2349.8 GBq/μmol) resulted in superior-quality images. Conversely, images from the probe with low molar activity (“LA_m_”, less than 172.5 GBq/μmol) showed poor PET and CLI signals in iBAT (**Figure 4B**). Quantitative analysis indicated that the PET signal in the HA_m_ group was 4.06-fold higher than in the LA_m_ group (“HA_m_” *vs.* “LA_m_”, 14.27 ± 3.28 *vs.* 3.51 ± 0.9, ^****^P < 0.0001, **Figure 4C**), and the CLI signal in the HA_m_ group was 1.54-fold higher than in the LA_m_ group (Average radiance, photon/s/cm^2^/sr, “HA_m_” *vs.* “LA_m_” 16328.57 ± 5781.78 *vs.* 10632.86 ± 1864.58, ^*^P = 0.04, **Figure 4D**). Our results indicated that probes with higher molar activity produced better iBAT images in TSPO-PET and CLI. In particular, a higher molar activity of the probe was required for quantitative analysis of CLI.

### Cold stimulation increases both TSPO-PET and TSPO-CLI signals in iBAT

**Figure 5:**
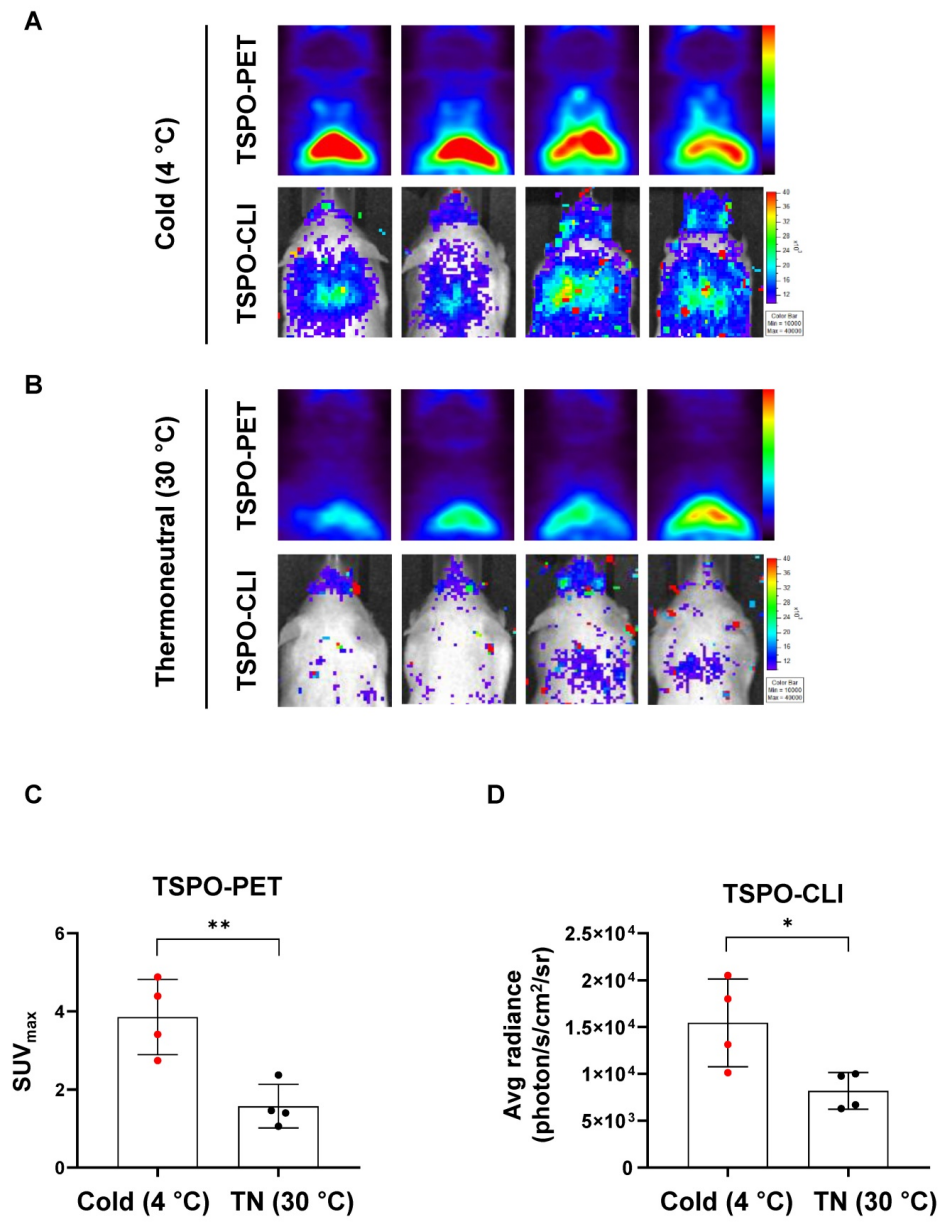
Cold stimulation significantly increases both TSPO-PET and TSPO-CLI signals in iBAT. (A) Representative TSPO-PET and TSPO-CLI images with [^18^F]fm-PBR28-*d*_2_ under cold stimulation (4 °C) for 4 h. (B) Representative TSPO-PET and TSPO-CLI images with [^18^F]fm-PBR28-*d*_2_ under thermoneutral condition (30 °C) for 4 h. (C) Quantitative analysis of PET signals from iBAT after [^18^F]fm-PBR28-*d*_2_ injection under cold stimulation (4 °C) or thermoneutral condition (30 °C). (D) Quantitative analysis of CLI signals from iBAT after [^18^F]fm-PBR28-*d*_2_ injection under cold stimulation (4 °C) or thermoneutral condition (30 °C) . Data represent means ± SD (n  = 4 per group). *P < 0.05, **P < 0.01.

In [^18^F]FDG-PET, the detection rate of iBAT is less than 10% in human, but cold stimulation increases the image detection rate of iBAT by 33~100% 32. Therefore, it is important to investigate and compare the effects of cold stimulation in iBAT imaging. We compared iBAT imaging by PET and CLI using TSPO-targeting probe under thermoneutral (TN, 30 °C) or cold stimulation (4 °C) conditions in UCP1 ThermoMouse. UCP1 ThermoMouse was exposed for 4 h to each group of TN or cold stimulation. **Figure 5A** shows the TSPO-PET signals from [^18^F]fm-PBR28-*d*_2_ in iBAT were much higher in the cold stimulation group (2.45-fold) than the thermoneutral condition group (SUV_max_, “cold” *vs.* “thermoneutral”, 3.86 ± 0.96 *vs.* 1.57 ± 0.56, ^**^P = 0.0064, **Figure 5A-B upper panel and 5C**). Similarly, TSPO-CLI signals from [^18^F]fm-PBR28-*d*_2_ in iBAT were significantly higher in the cold stimulation group (1.86-fold) than the thermoneutral condition group (Average radiance, photon/s/cm^2^/sr, “cold” *vs.* “thermoneutral”, 15437.5 ± 4683.15 *vs.* 8259.5 ± 2043.58, ^*^P = 0.029, **Figure 5A-B lower panel and 5D**). Consistent with previous other reports, [^18^F]FDG-PET (1.53-fold) and [^18^F]FDG-CLI (1.18-fold) signals from [^18^F]FDG in iBAT were higher in the cold stimulation group (**Figure S1**). However, our data suggested that TSPO-targeting probe was more sensitive and specific reflected in iBAT imaging than using [^18^F]FDG.

### Short isoflurane exposure shows higher both TSPO-PET and TSPO-CLI signals than long isoflurane exposure

**Figure 6:**
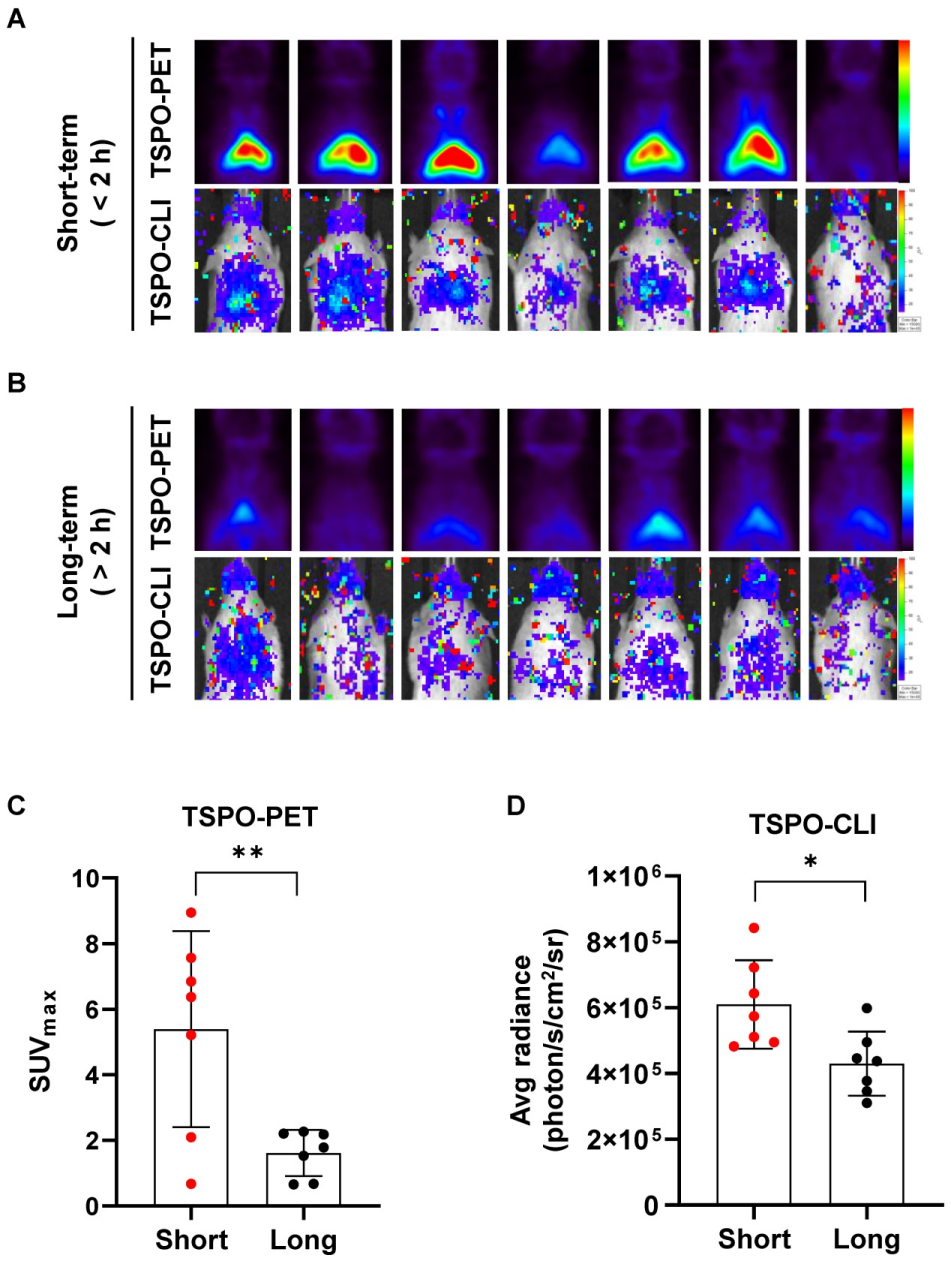
Short isoflurane exposure shows higher both TSPO-PET and TSPO-CLI signals than long isoflurane exposure in iBAT. (A) Representative TSPO-PET and TSPO-CLI images with [^18^F]fm-PBR28-*d*_2_ under short exposure of isoflurane anesthesia (“short-term”, shorter than 2 h). (B) Representative TSPO-PET and TSPO-CLI images with [^18^F]fm-PBR28-*d*_2_ under long exposure of isoflurane anesthesia (“long-term”, longer than 2 h). (C) Quantitative analysis of PET signals from iBAT after [^18^F]fm-PBR28-*d*_2_ injection under short or long exposure of isoflurane anesthesia. (D) Quantitative analysis of CLI signals from iBAT after [^18^F]fm-PBR28-*d*_2_ injection under short or long exposure of isoflurane anesthesia. Data represent means ± SD (n  = 7 per group). *P < 0.05, **P < 0.01.

We compared iBAT activity changes following prolonged anesthesia by PET and CLI using a TSPO-targeting probe. UCP1 ThermoMouse was anesthetized with isoflurane for less than 2 h (short-term) or more than 2 h (long-term) prior to intravenous injection of [^18^F]fm-PBR28-*d*_2_ (**Figure 6A-B**). TSPO-PET signals from [^18^F]fm-PBR28-*d*_2_ in iBAT were much higher in the short-term (3.3-fold) than the long-term exposure group (SUV_max_, “short-term” *vs.* “long-term”, 5.387 ± 2.989 *vs.* 1.613 ± 0.699, ^**^P = 0.0069, **Figures 6A-B upper panel and 6C**). Similarly, TSPO-CLI signals from [^18^F]fm-PBR28-*d*_2_ in iBAT were significantly higher in the short-term (1.41-fold) than the long-term exposure group (Average radiance, photon/s/cm^2^/sr, “short-term” *vs.* “long-term”, 609857.1 ± 134265 *vs.* 429857.1 ± 97484.1, ^*^P = 0.0141, **Figure 6A-B lower panel and 6D**). Interestingly, in the case of [^18^F]FDG, we found no significant difference in the PET and CLI imaging between the two groups with different exposure times to anesthesia (**Figure S2**). Our data showed that [^18^F]fm-PBR28-*d*_2_ produced a more sensitive and specific iBAT image than [^18^F]FDG by better reflecting UCP1 expression and providing enough CLI signal to evaluate the iBAT activity, suggesting that TSPO-CLI could be used as an alternative imaging technique to TSPO-PET for iBAT imaging.

## Discussion

Recently, adipose tissue (AT) imaging has been spotlighted to diagnose and treat various metabolic diseases. Obesity is assessed by the amount of whole-body AT using body mass index (BMI) or the thickness of white adipose tissue by computed tomography (CT). Besides the white adipose tissue, the assessment of obesity requires a more detailed analysis of other adipose tissue types, such as the brown adipose tissue (BAT). Generally, iBAT images are acquired by cold stimulation or chemical treatments such as rosiglitazone and β-3 adrenergic agonist using [^18^F]FDG-PET 5, 6. However, due to the elevated uptake of [^18^F]FDG in tissues with high glucose metabolism, such as the heart and brain 10, [^18^F]FDG-PET quantitative analysis of iBAT is not reliable.

The UCP1 ThermoMouse is a valuable model to image iBAT as it specifically shows the expression of endogenous UCP1, a biomarker of BAT, by bioluminescence and fluorescence imaging *in vivo* 15. We validated high UCP1 and TSPO expression in iBAT using the UCP1 ThermoMouse (**Figures 1 and 2**). We evaluated the biodistribution of [^18^F]FDG and [^18^F]fm-PBR28-*d*_2_* in vivo* and observed specific uptake of [^18^F]fm-PBR28-*d*_2_ in iBAT. Since [^18^F]FDG is capable of targeting iBAT, PET and CLI images are limited by changes in glucose metabolism in the body, its high uptake in the brain and heart could interfere with the reliability of [^18^F]FDG in iBAT images. We tested the feasibility of [^18^F]FDG and [^18^F]fm-PBR28-*d*_2_ for iBAT imaging in UCP1 ThermoMouse and we divided them into two groups (“high” and “low”) according to their UCP1 levels. We found that the [^18^F]fm-PBR28-*d*_2_ uptake correlated with endogenous levels of UCP1. However, we did not detect a correlation between the [^18^F]FDG uptake and endogenous levels of UCP1 (**Figure 3**). These data indicated that [^18^F]fm-PBR28-*d*_2_ was significantly more useful than [^18^F]FDG for obtaining iBAT images in both PET and CLI.

Because the detection rate of iBAT in [^18^F]FDG-PET is less than 10% in human, there is inconvenient to acquire images of iBAT by stimulation using cold climate or β-3 adrenergic receptor agonist 32. In addition to higher uptake by brain and heart, skeletal muscle uptake may also interfere with obtaining iBAT-specific images. Therefore, its location has been verified by combining with anatomical images such as CT or magnetic resonance angiography (MRI). It was reported that glucose uptake in iBAT is about 8-fold higher than skeletal muscles but the total amount of iBAT in the whole body is only 0.2% of skeletal muscles 50. Therefore, the amount of total glucose uptake in iBAT is only about 1% of that of skeletal muscles. On the other hand, both TSPO and UCP1 proteins are present in mitochondria, and the brown color of BAT is derived from a smaller droplet size with a higher number of mitochondria compared to WAT, so TSPO is considered to represent the expression of UCP1 relatively well.

Since an optimal dose of radionuclide activity is required for an accurate PET scan 31, the molar activity of an imaging probe A_m_, defined as the radioactivity in a specific amount of tracer, is important 33. We determined that higher molar activity was a significant factor for CLI and PET. Especially for [^18^F]fm-PBR28-*d*_2_, a molar activity of at least 2300 GBq/μmol was required to obtain better CLI images (**Figure 4**). However, too high molar activity might saturate probe uptake in the target organ, making accurate observation difficult. Therefore, it was necessary to determine the optimal range of molar activity of a probe to obtain high-quality images 34. Although PET instruments effectively visualize and quantify the uptake of radioactive probes in non-invasive whole-body imaging, their cost is prohibitive for routine use. Since most optical imaging equipment is less expensive than PET equipment, CLI imaging using Cerenkov radiation from a PET probe can be used as an alternative. Therefore, we tested the comparability of PET and CLI images for iBAT in the UCP1 ThermoMouse model. Our results showed that TSPO-PET with [^18^F]fm-PBR28-*d*_2_ reflected UCP1 expression in iBAT better than [^18^F]FDG-PET and was more sensitive for iBAT imaging. In addition, iBAT images could be obtained with CLI using [^18^F]fm-PBR28-*d*_2_, and TSPO-CLI could be used as an alternative imaging technique to TSPO-PET for iBAT imaging.

From 2003 to 2012, since the detection rate of iBAT in [^18^F]FDG-PET is less than 10% in human but cold stimulation increases the image detection rate of iBAT for 33~100% 32. As we mentioned above, UCP1 expression increases in iBAT that uncouples the respiratory chain from oxidative phosphorylation resulting high rate of oxidation and capable of using metabolic energy expenditure to provide heat under cold stimulation 2, 7, 35. we showed that TSPO-PET and TSPO-CLI of signals in iBAT with [^18^F]fm-PBR28-*d*_2_ were significant higher in the cold stimulation group than thermoneutral condition group (**Figure 5**). Also, the correlation between UCP1 expression and [^18^F]FDG uptake under cold stimulation condition in iBAT was evaluated. (**Figure S1**). However, these results suggest that more sensitive and specific iBAT imaging is possible using TSPO-targeting probe than [^18^F]FDG.

General anesthesia plays an important role in the long-term PET imaging and patient surgery in the clinic and is also essential in animal studies 36, 37. Effects of anesthetics on mitochondrial metabolism have been extensively studied, suggesting that anesthesia has a detrimental effect on mitochondrial function 38. For example, isoflurane was shown to decrease the binding of [^11^C]DPA-713, a TSPO radioligand, in the monkey brain 39. These data suggested that anesthetics may affect the TSPO binding affinity differently, acting as a variable for TSPO-PET imaging. Indeed, we observed that the binding of [^18^F]fm-PBR28-*d*_2_ in PET and CLI was decreased in the group with longer exposure to anesthesia than with shorter exposure (**Figure 6**). On the other hand, UCP1 expression and [^18^F]FDG uptake in iBAT of UCP1 ThermoMouse did not change even after “long term” isoflurane exposure compared to “short-term” exposure (**Figure S2**). These data indicated that anesthetics might decrease the metabolic function of mitochondria, thereby inhibiting TSPO expression or function and consequently affecting iBAT imaging. However, iBAT images using [^18^F]fm-PBR28-*d*_2_ are generally acquired within 1 h and may not be influenced by the long-term anesthesia effect.

In addition to the results of our research in this paper, various iBAT studies are possible in UCP1 ThermoMouse using [^18^F]FDG-PET or TSPO-PET probes. The iBAT images obtained from human [^18^F]FDG-PET showed decreased glucose uptake with age due to mitochondrial dysfunction and impairment of endocrine signaling 40-44, suggesting that TSPO-PET may also be affected by age. Preliminary, we observed that a young UCP1 ThermoMouse (9 weeks old) had higher signals in both TSPO-PET and TSPO-CLI than an older UCP1 ThermoMouse (36 weeks old) under normal condition (**Figure S3**). However, additional experiments are needed to explain this observation. Also, since most obese people have white fat as adipocytes, research on obesity treatment through browning of white fat is of interest. It is well established that diet-induced obese (DIO) mouse models or obese humans have larger adipocytes, fewer mitochondria, and lower UCP1 expression than lean mice or humans 35, 45-49. Therefore, iBAT images with UCP1 and TSPO-targeting ligands in the DIO model of UCP1 ThermoMouse are expected to provide various information on browning of white fat.

## Conclusions

In this study, we revealed that the TSPO-targeting probe, [^18^F]fm-PBR28-*d*_2,_ can be used as a reliable iBAT imaging probe that reflects UCP1 expression. We also confirmed that [^18^F]fm-PBR28-*d*_2_ can be used for TSPO-PET and TSPO-CLI to visualize iBAT. Significantly, our results showed that the TSPO-CLI can effectively replace TSPO-PET under defined conditions.

## Materials and Methods

### Animals

All experiments were approved by the Institutional Animal Care and Use Committee of the Seoul National University (IACUC No. 18-1228-2, 28 December 2018). UCP1 ThermoMouse (UCP1-Luc2-tdTomato reporter transgenic mouse) were obtained from Jackson Laboratory (ME, USA). Only 8-16 weeks old males were used for this research study because the reporter transgene was inserted into the Y chromosome. Carrier males were mated with FVB/NJ female mice to maintain live colonies. Offspring were identified by genotyping with *Ucp1* primer. Sequences of the primers to validate the UCP1 reporter gene insertion were as follows. 5'-GTGCCACTGTTGTCTTCAGG-3' (forward); 5'-ATAGCTTCTGCCAACCGAAC-3' (reverse).

### Immunohistochemisty (IHC)

Tissues from UCP1 ThermoMice were dissected and fixed with 4% paraformaldehyde. Fixed tissues were paraffin-embedded and cut with a microtome to generate 4 μm slices. Subsequently, tissue slices were mounted on the glass slides. For the IHC analysis, slides were deparaffinized, rehydrated, and treated with 0.5% H_2_O_2_ (386790, Calbiochem, San Diego, CA, USA) for 30 min for endogenous peroxidase blocking. The slides were boiled with 10 mM sodium citrate (pH 6.0) (ICN biomedicals, Aurora, OH, USA) for antigen retrieval and permeabilized with 0.5% Triton X-100 (35501, Yakuri pure chemicals, OSAKA, Japan) in Tris-buffered saline (TBS) for 5 min. Antibodies were diluted with TBS containing 1% BSA and incubated for 24 h at 4 ºC. Primary antibodies included rabbit isotype control (02-6102, Invitrogen, Waltham, MA, USA), goat isotype control (31245, Invitrogen, Waltham, MA, USA), anti-UCP1 (ab10983, Abcam, Cambridge, UK), anti-luciferase (NB100-1677, Novus Biologicals, Centennial, CO, USA), and anti-TSPO (ab109497, Abcam, Cambridge, UK). Immunolabeling was performed with biotinylated horse anti-rabbit IgG (BA-1100, Vector Laboratories, Burlingame, California, USA) and horse anti-goat IgG (BA-9500, Vector Laboratories, Burlingame, California, USA) secondary antibodies for 1 h followed by binding with ABC (PK-6100, Vector Laboratories, Burlingame, California, USA) for 1 h. Finally, 3, 3'-diaminobenzidine (DAB) (SK-4100, Vector Laboratories) was used as a chromogenic substrate (SK-4100, Vector Laboratories), and slides were counterstained with hematoxylin.

### Western blotting

Extracted proteins from eWAT, iWAT, epididymis, iBAT, testis and whole brain (10 μg) were loaded and separated on bis-Tris-HCl buffered 10% sodium dodecyl sulfate (SDS)-polyacrylamide gels. Separated proteins were transferred to polyvinylidene fluoride (PVDF) membranes and incubated at 4 ºC with anti-UCP1 (ab10983, Abcam, Cambridge, UK), anti-TSPO (ab109497, Abcam, Cambridge, UK), and anti-β-actin (A5441, Sigma-Aldrich, St. Louis, MO, USA)] antibodies for overnight. Next, membranes were incubated with horseradish peroxide-linked goat anti-rabbit (7074S, Cell Signaling Technology, Danvers, MA, USA) and horse anti-mouse (7076S, Cell Signaling Technology, Danvers, MA, USA) secondary antibodies for 1 h at room temperature. All immunoreactive bands were imaged with an LAS-3000 imaging system (Bio-Rad Laboratories, Hercules, CA, USA).

### Ex vivo biodistribution of PET probes

Radioactive [^18^F]fluorodeoxyglucose ([^18^F]FDG) and deuterium-substituted [^18^F]fluoromethyl-PBR28 ([^18^F]fm-PBR28-*d*_2_) were synthesized following the protocols described previously 26. UCP1 ThermoMouse underwent intravenous injection with [^18^F]FDG or [^18^F]fm-PBR28-*d*_2_. After 1 h, mice were euthanized, and tissue samples were collected, including blood, muscle, bone, intestine, stomach, testes, spleen, kidney, liver, heart, lung, iWAT, eWAT, and iBAT. Radioactivity of the samples was measured by Cobra II gamma counter (Canberra Packard; Vaughan, Ontario, Canada). Results were expressed as the percentage of injected dose per gram (%ID/g).

### Bioluminescence imaging

Bioluminescence imaging was acquired using an IVIS 100 imaging system (Xenogen, Alameda, CA, USA). UCP1 ThermoMice were anesthetized with 1.5% isoflurane combined with oxygen. Subsequently, D-luciferin (3 mg per mouse, #E1605, Promega, WI, USA) was injected intraperitoneally, and bioluminescence signals were obtained and analyzed with Living Imaging software (ver.2.50.2, Xenogen, Alameda, CA, USA).

### Cerenkov luminescence imaging

CLI was acquired using an IVIS 100 imaging system. UCP1 ThermoMice were anesthetized with isoflurane and intravenously injected with [^18^F]FDG (11.1 to 14.8 MBq per mouse) or [^18^F]fm-PBR28-*d*_2_ (11.1 to 14.8 MBq per mouse). Mice were then subjected to CLI sequentially for 1 h after [^18^F]FDG or [^18^F]fm-PBR28-*d*_2_ injection with the following parameters: open filter, f/stop = 1, bin = 4, FOV = D, and exposure time = 300 s.

### PET imaging

PET scans were acquired by small animal PET imaging (SimPET, Brightonics imaging, Seoul, Korea) to visualize iBAT. Mice were anesthetized with 1.5% isoflurane combined with oxygen during the PET scans. PET images were acquired for 10 min and reconstructed by using the 3D OSEM algorithm (12 subsets). PET images were reconstructed and analyzed by the AMIDE program (ver. 0.9.0, http://amide.sourceforge.net). An ellipsoidal region-of-interest (ROI) was drawn around the target on the iBAT and expressed as a standardized uptake value (SUV) in a sagittal or a coronal PET slice using the AMIDE program (ver. 0.9.0, http://amide.sourceforge.net). Subsequently, the maximum PET SUV ratio in iBAT was calculated.

### Statistical analysis

All results were calculated as mean ± standard deviation (SD), and statistical significance was determined using the unpaired 2-sample parametric Student t-test. GraphPad Prism 8 software (GraphPad Software Inc., San Diego, CA, USA) was used for statistical analysis. *P* < 0.05 was considered statistically significant.

## Supplementary Material

Supplementary figures.

## Figures and Tables

**Table 1:** Biodistribution of [^18^F]FDG *vs.* [^18^F]fm-PBR28-*d*_2_.

	[^18^F]FDG (%ID/g)		[^18^F]fm-PBR28-*d*_2_ (%ID/g)			P-value
Organs	Mean	SD	Mean	SD		
Blood	1.67	0.47	1.86	0.67		
Muscle	1.16	0.44	1.34	0.11		
Bone	2.08	0.42	2.34	0.2		
Intestine	3.61	0.60	5.08	0.71		
Stomach	2.14	0.79	3.31	0.68		
Testis	3.96	0.20	1.46	0.20		
Spleen	4.06	0.52	17.01	4.44		
Kidney	16.38	10.91	38.19	14.21		
Liver	2.33	0.75	3.18	1.16		
Heart	56.74	10.6	15.88	13.6	*	
Lung	6.19	1.09	31.66	19.63		
iWAT	1.78	0.08	5.21	1.55		
eWAT	0.33	0.08	1.87	0.81		
iBAT	7.68	2.47	34.08	6.32	**	
Brain	6.62	1.38	1.52	0.96	**	
						

*P < 0.05, **P < 0.01.

## Abbreviations

UCP1: uncoupling protein 1
[^18^F]FDG: [^18^F]fluorodeoxyglucose
TSPO: translocator protein-18 kDa
PET: positron emission tomography
AT: Adipose tissue
BAT: brown adipose tissue
iBAT: interscapular brown adipose tissue
WAT: white adipose tissue
iWAT: inguinal white adipose tissue
eWAT: epididymal white adipose tissue
BLI: bioluminescence imaging
CLI: Cerenkov luminescence imaging
CR: Cerenkov radiation
BAC: Bacterial artificial chromosome
Luc2: Luciferase-2
A_m_: molar activity
TN: thermoneutral
ROI: region of interest
SUV: standardized uptake value
CT: Computed tomography
MRI: Magnetic resonance angiography
