# Supplementary material for: Cerenkov luminescence imaging of interscapular brown adipose tissue using a TSPO-targeting PET probe in the UCP1 ThermoMouse
Source: Theranostics. 2022 Aug 29;12(14):6380–94. doi: 10.7150/thno.74828 (PMC9475450; doi:10.7150/thno.74828)
Supplement: Supplementary file 1 — Supplementary figures. [file thnov12p6380s1.pdf]

Cerenkov luminescence imaging for visualizing interscapular brown adipose tissue using a TSPO-targeting PET probe in UCP1 ThermoMouse (Supporting information)

Figure S1. Cold stimulation also increases both [^{18}F]FDG-PET and [^{18}F]TSPO-CLI signals in iBAT. (A) Representative [^{18}F]FDG-PET and [^{18}F]FDG-CLI images under cold stimulation (4 °C) for 4 h. (B) Representative [^{18}F]FDG-PET and [^{18}F]FDG-CLI images under thermoneutral condition (30 °C) for 4 h. (C) Quantitative analysis of PET signals from iBAT after [^{18}F]FDG injection under cold stimulation (4 °C) or thermoneutral condition (30 °C). (D) Quantitative analysis of CLI signals from iBAT after [^{18}F]FDG injection under cold stimulation (4 °C) or thermoneutral condition (30 °C). Data represent means ± SD (n = 4 per group). *$P < 0.05$.

Figure S2. Isoflurane exposure does not affect signals of [^{18}F]FDG-PET and [^{18}F]FDG-CLI on iBAT. (**A**) Representative [^{18}F]FDG-PET and [^{18}F]FDG-CLI images under short exposure of isoflurane anesthesia ("short-term", shorter than 2 h). (**B**) Representative [^{18}F]FDG-PET and [^{18}F]FDG-CLI images under long exposure of isoflurane anesthesia ("long-term", longer than 2 h). (**C**) Quantitative analysis of PET signals from iBAT after [^{18}F]FDG injection under short or long exposure of isoflurane anesthesia. (**D**) Quantitative analysis of CLI signals from iBAT after [^{18}F]FDG injection under short or long exposure of isoflurane anesthesia. Data represent means ± SD (n = 4 per group). ns, not significant.

Figure S3. Young UCP1 ThermoMouse shows higher signals of TSPO-PET and TSPO-CLI than older UCP1 ThermoMouse. Representative images using TSPO-PET and TSPO-CLI in young UCP1 ThermoMouse (9 weeks old) and older UCP1 ThermoMouse (36 weeks old) under normal condition. (n = 1 per group).

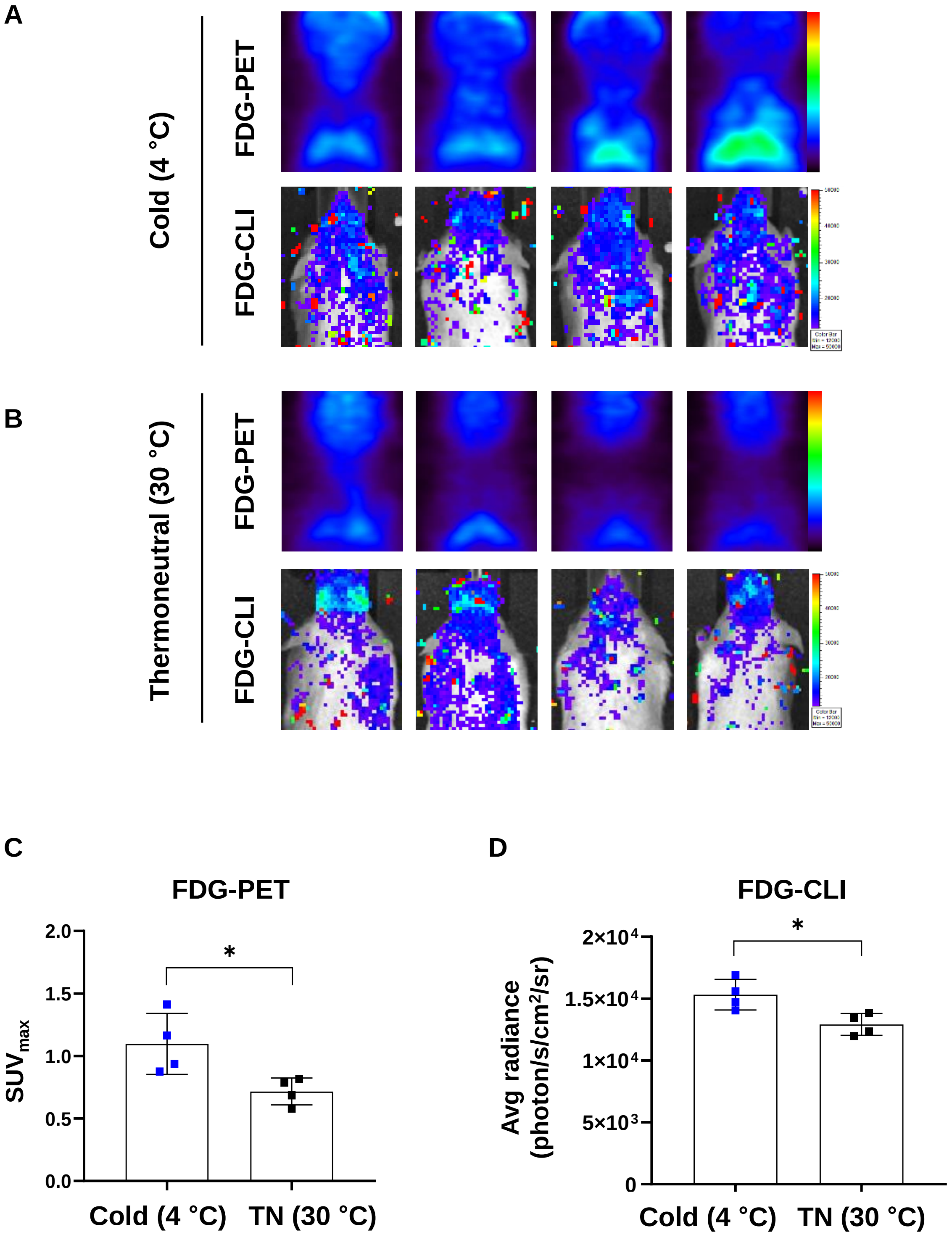
A
Cold (4 °C)
FDG-PET
FDG-CLI
B
Thermoneutral (30 °C)
FDG-PET
FDG-CLI
C
FDG-PET
SUVmax
2.0
1.5
1.0
0.5
0.0
*
Cold (4 °C)
TN (30 °C)
D
FDG-CLI
Avg radiance (photon/s/cm2/sr)
2×10^4
1.5×10^4
1×10^4
5×10^3
0
*
Cold (4 °C)
TN (30 °C)

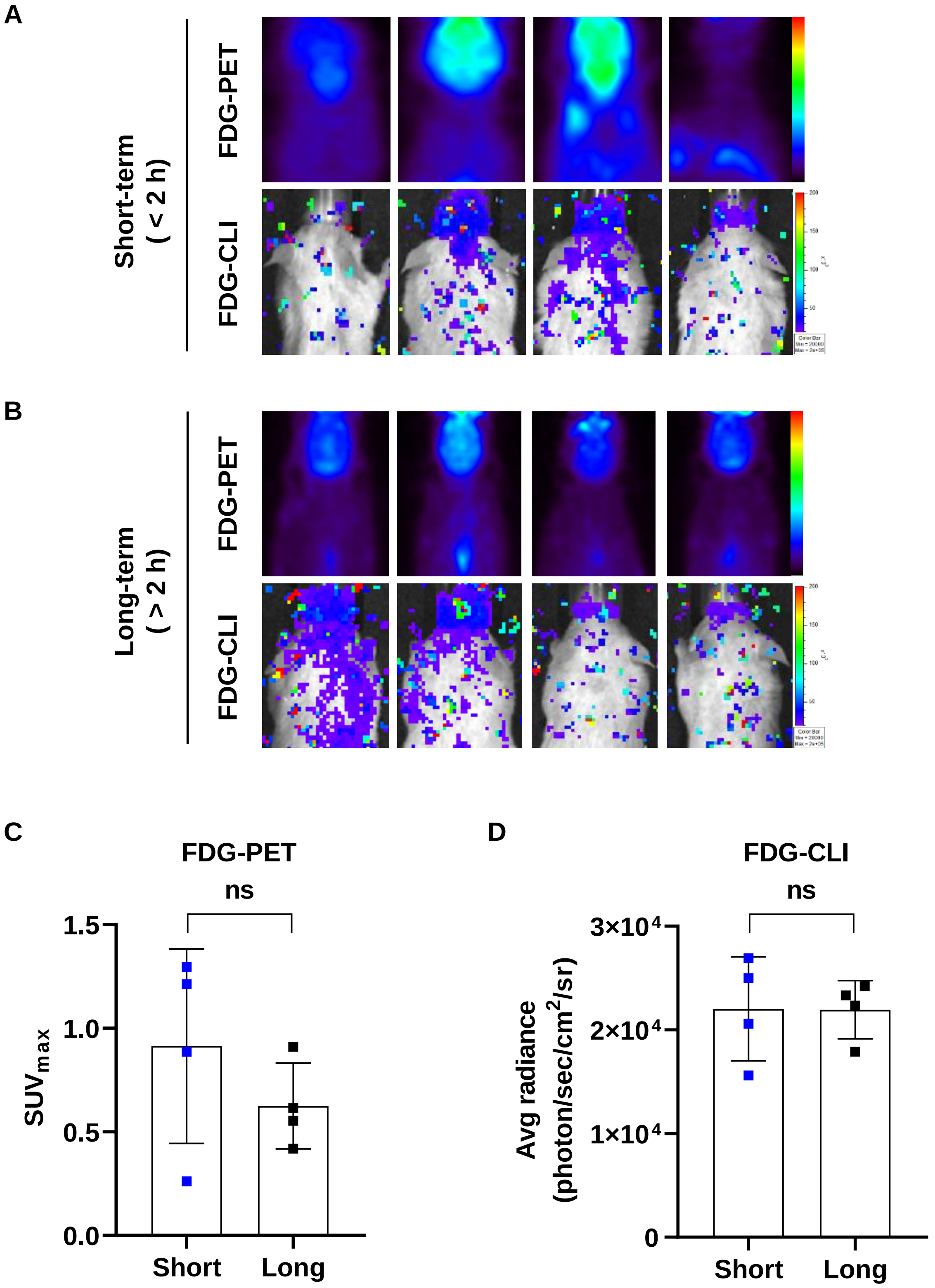
A
Short-term
(< 2 h)
FDG-PET
FDG-CLI
B
Long-term
(> 2 h)
FDG-PET
FDG-CLI
C
FDG-PET
ns
SUV_{max}
1.5
1.0
0.5
0.0
Short
Long
D
FDG-CLI
ns
Avg radiance
(photon/sec/cm^2/sr)
3×10^4
2×10^4
1×10^4
0
Short
Long

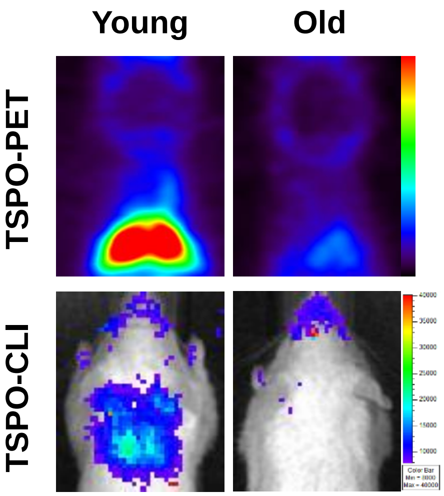
Young
Old
TSPO-PET
TSPO-CLI
40000
35000
30000
25000
20000
15000
10000
Color Bar
Min = 8000
Max = 40000
